# A structural equation model of the relationship between insomnia, negative affect, and paranoid thinking

**DOI:** 10.1371/journal.pone.0186233

**Published:** 2017-10-19

**Authors:** Alexander J. Scott, Georgina Rowse, Thomas L. Webb

**Affiliations:** 1 School of Health and Related Research (ScHARR), University of Sheffield, Sheffield, United Kingdom; 2 Clinical Psychology Unit, University of Sheffield, Sheffield, United Kingdom; 3 Department of Psychology, University of Sheffield, Sheffield, United Kingdom; Neurocenter of Southern Switzerland, SWITZERLAND

## Abstract

**Background:**

A growing body of evidence points to relationships between insomnia, negative affect, and paranoid thinking. However, studies are needed to examine (i) whether negative affect mediates the relation between insomnia and paranoid thinking, (ii) whether different types of insomnia exert different effects on paranoia, and (iii) to compare the impact of objective and self-reported sleeping difficulties.

**Method:**

Structural equation modelling was therefore used to test competing models of the relationships between self-reported insomnia, negative affect, and paranoia. *n* = 348 participants completed measures of insomnia, negative affect and paranoia. A subset of these participants (*n* = 91) went on to monitor their sleep objectively (using a portable sleep monitor made by Zeo) for seven consecutive nights. Associations between objectively recorded sleep, negative affect, and paranoia were explored using linear regression.

**Results:**

The findings supported a fully mediated model where self-reported delayed sleep onset, but not self-reported problems with sleep maintenance or objective measures of sleep, was directly associated with negative affect that, in turn, was associated with paranoia. There was no evidence of a direct association between delayed sleep onset or sleep maintenance problems and paranoia.

**Conclusions:**

Taken together, the findings point to an association between perceived (but not objective) difficulties initially falling asleep (but not maintaining sleep) and paranoid thinking; a relationship that is fully mediated by negative affect. Future research should seek to disentangle the causal relationships between sleep, negative affect, and paranoia (e.g., by examining the effect of an intervention using prospective designs that incorporate experience sampling). Indeed, interventions might profitably target (i) perceived sleep quality, (ii) sleep onset, and / or (iii) emotion regulation as a route to reducing negative affect and, thus, paranoid thinking.

## Introduction

Sleep problems and mental health complaints go hand-in-hand, with problems sleeping associated with many, if not all, psychiatric conditions [1–4]. This association is particularly apparent in the experience of paranoia—a person’s belief “that harm is occurring, or is going to occur, to him or her” and “that the persecutor has the intention to cause harm” [5]. West, Cornelis, Janszen, and Lester [6] commented that, of the symptoms which often accompany sleep deprivation, “gross delusional thinking, usually paranoid, becomes increasingly prominent” (p. 69), with subsequent empirical research providing additional evidence on the association [7–11]. The present research focuses on one particular sleep problem; namely, the experience of insomnia (defined as difficulties initiating and/or maintaining sleep), and its relationship with paranoid thinking. Both insomnia and paranoia are relatively common in the general population. For example, Ohayon reported that around one third of people experience at least one symptom of insomnia according to DSM-IV criteria [12–15]. The experience of paranoia is also common, with numerous studies reporting incidences of paranoid thinking. For example, Freeman et al. [16] found that approximately one third of a sample of 1,202 people from the general population reported experiencing paranoid thoughts (see also, [17, 18, 19]). It is therefore crucial to understand whether sleep problems are related to paranoia and, if so, why.

### The relationship between insomnia and paranoia: What do we know?

Problems sleeping have been integrated into current theoretical models of the development of paranoia. For example, Freeman et al. [20] propose that the formation paranoid thoughts is underpinned by an “interaction between vulnerability (from genetic, biological, psychological, and social factors) and stress (which may also be biological, psychological, or social)” (p. 333, [20]. In this model, the formation of a paranoid belief starts with a precipitator (e.g., a stressful life event or occurrence) which leads to increased arousal that can be exacerbated by other factors such as sleep disturbances and affective experiences. In an updated account of Freeman et al’s [20] theoretical model, Freeman [21] proposes that problems sleeping contribute to the experience of paranoia via multiple routes, including elevating negative emotion, mood dysregulation and anomalous experiences, as well as impairing cognitive ability to appraise interpretations of ambiguous situations.

Several empirical studies have investigated the relationship between insomnia and the experience of paranoia. For example, a strong association between insomnia and paranoia has been reported in both community samples (i.e., among those with no history of mental illness) and clinical samples (i.e., among those with a diagnosis of psychosis) [9]. The British National Survey of Psychiatric Morbidity [22] provided further evidence of an association between insomnia and paranoia, finding that insomnia was associated with an increase in the frequency of paranoid thoughts [8]. Interestingly, both Freeman et al. studies [8, 9] found a weaker relationship between insomnia and paranoia when levels of depression and anxiety were taken into account, suggesting that the relationship may be explained, in part, by increases in negative affect.

Research that employs prospective designs supports the idea that insomnia and negative affect contribute to the experience of paranoia. For example, insomnia, worry, anxiety, and depression were all found to predict new and persisting paranoid thinking in a longitudinal study [10]. Studies have also used experience sampling to investigate the link between sleep, affect, and paranoid thinking (for an overview, see [23]). For example, Mulligan et al. [24] found that more fragmented sleep and poorer self-reported sleep quality were associated with more frequent paranoid experiences in a sample of 22 participants with a diagnosis of schizophrenia. Furthermore, as in previous research, this effect was found to be mediated by negative affect on awakening. Subsequent research using experience sampling with adolescents has found that levels of paranoia could be predicted by the total time spent asleep on the previous night and the frequency of dreaming; an effect that, again, was partially mediated by affective experiences [25]. Finally, Reeve, Emsley, Sheaves and Freeman [26] compared a group of non-clinical participants whose sleep had been restricted to a group with no sleep restriction and subsequently investigated the impact on psychotic experiences and candidate mediating mechanisms (i.e., negative affect, cognition and perceptual processing). The authors reported that those in the sleep restriction condition reported significant increases in psychosis like experiences (including paranoia and hallucinations), an effect mediated by changes in negative affect and related processes. In summary, insomnia appears to be associated with the experience of paranoia in both the general population and in clinical samples. Furthermore, this association is likely to be mediated to some degree by affective experiences; particularly, levels of depression, stress, and anxiety.

### The role of affective experiences in the relationship between insomnia and paranoia

The evidence above suggests that negative affect plays an important role in the relationship between insomnia and paranoia, mediating (at least some of) the effects of insomnia on the experience of paranoia. This is perhaps not surprising given the established links between; i) insomnia and negative affect; and ii) between negative affect and paranoia. For example, Taylor et al. [27] reported that, in the presence of insomnia, participants were 9.82 and 17.35 times more likely to have clinically meaningful levels of depression and anxiety, respectively. Furthermore, problems sleeping have been shown to be predictive of negative affect in a recent meta-analysis of longitudinal studies [28]. There is also evidence that the severity of affective experiences is reduced when sleep is improved through intervention [29–33].

The relationship between negative affect and the experience of paranoia is also well documented. For example, Kramer et al. (2014) used experience sampling to examine the moment-to-moment interplay between negative affect and the experience of paranoia, reporting that increases in negative affect were associated with increases in paranoia (see also, [34]). Other researchers have experimentally manipulated affective experiences and then compared participants’ performance on proxy measures of paranoia, such as The Beads Task (a proxy measure of paranoia, see [35]. The typical finding is that those induced to experience negative affect reported significantly more paranoid thoughts and a more pronounced Jumping to Conclusions bias (JTC, for a review see [36] when compared to participants who were not induced to experience negative affect [35, 36]. Finally, structural equation modelling of longitudinal data has found that depressed mood is causally related to paranoid thoughts; an effect that is mediated by negative cognition [37].

### What are the limitations of the extant literature?

Despite evidence pointing to relationships between sleep, affect, and paranoia, there are several methodological weaknesses with the existing evidence base that limit what can be concluded. First, to date, the relationship between sleep and paranoia has largely been investigated using self-report measures of insomnia (for notable exceptions, see [24, 25]. However, discrepancies between self-report and objective measures of sleep are common [38–42] and there is a tendency for people with insomnia to overestimate how long it takes them to get to sleep and also to underestimate the total amount of time that they spend asleep, relative to objective measures [43]. Given that self-report and objective measures of difficulties sleeping may differ, it would be valuable to investigate whether actual or perceived sleep difficulties (or both) are associated with paranoia.

Second, previous research has tended to use relatively limited measures of paranoia. For example, studies have used only two items from the Psychosis Screening Questionnaire to capture the experience of paranoia [8, 10]. As Freeman et al. [10] pointed out, these questions “provided a limited capture of the variety of paranoid ideation” (p. 1202). There are some exceptions—for example, Grezellschak et al. [15] used multi-item measures of both insomnia (i.e., the Insomnia Severity Index, [44] and paranoid thinking (i.e., the Paranoia Checklist, [16]; reporting that the association between insomnia and paranoid ideation is partially mediated by emotion regulation. However, Grezellschak et al., did not include an objective measure of sleep nor did they investigate the independent contribution of sleep onset and maintenance difficulties.

Third, as suggested above, insomnia has so far been conceptualised as a single construct in research on the relationship between sleep problems and paranoia. However, problems with sleep onset and sleep maintenance (two distinctive facets of insomnia, [12] may be differentially related to affective experiences and paranoia. For example, Sheaves et al. [45] reported that delayed sleep onset latency was more prevalent than sleep maintenance problems in those at high risk of a mental health problem in a sample of 1403 students (although onset and maintenance were assessed using only 1 item each). Delayed sleep onset can provide an opportunity for an individual to reflect, ruminate, and worry about their experiences [46–49], which could serve to amplify negative affect (for a review, see [50] and may even lead to paranoid thinking. The association between sleep maintenance problems and these experiences is less clear (i.e., either a weaker relationship or no relationship, see [49, 51–53]).

There are also unresolved conceptual issues—specifically, it is unclear whether insomnia has a direct impact on paranoia, or whether negative affect mediates (at least some of) the effects of insomnia on paranoia [7]. Sleep problems are often associated with negative affect [27] and have been shown to predict negative affect more generally [28]. Thus, one explanation for the relationship between insomnia and paranoia is that difficulties initiating and/or maintaining sleep lead to negative affect that, in turn, contribute to the formation and maintenance of paranoid thinking (a fully mediated model). Alternatively, insomnia could be directly associated with paranoid thinking. It is also important to note that the possibility of direct (unmediated) effects do not preclude the possibility that insomnia also has an indirect effect through negative affect as has been reported in previous research (i.e., a partially mediated model) [8–10].

### The present research

Evidence suggests that psychotic symptoms [54–56], and specifically paranoia [16], are not only confined to clinically defined groups, but are distributed across the general population. Thus, research studying aspects of the experience of psychosis among members of the general population can inform our understanding of the same experiences in clinical populations [55]. Consequently, the present research reports a cross-sectional, exploratory investigation into the associations between insomnia, negative affect, and paranoia in a sample drawn from the general population. The research aims to address limitations in the extant literature by; i) exploring whether objective indices of difficulties sleeping are associated with negative affect and paranoia; ii) improving the measurement of insomnia and paranoia by using multi-item measures with established reliability and validity and by conducting exploratory and confirmatory factor analyses; and iii) investigating direct, fully, and partially mediated models of the relationship between insomnia, negative affect, and paranoia using structural equation modelling (or SEM). It is hypothesised that; i) self-reported difficulties sleeping (and particularly, problems getting to sleep) will have both a direct association with paranoid experiences and an indirect association via negative affect; and ii) there will be a similar association between objectively recorded sleep and both negative affect and the experience of paranoia.

## Method

### Participants

The recommendations of Westland [57] were used to determine that the minimum sample size required for the intended SEM analyses to detect medium sized relationships with 80% power was *N* = 241. Based on the recommendations of Cohen, Cohen, West, and Aiken [58], a minimum sample size of *N* = 54 would be required to detect medium-sized relationships with 80% power using linear regression (our intended method for assessing the impact of objectively measured sleep problems on negative affect and paranoia). Consequently, *N* = 389 participants were recruited via an email to a list of volunteers at a large University in the UK. Of these, *N* = 41 (11%) did not complete the baseline measures, leaving *N* = 348 complete responses. These participants were aged between 18 and 77 (*M* = 36.49, *SD* = 12.76) and 264 (76%) of them were female. As the majority of the sample was female, MANOVA was used to investigate any associations between gender, insomnia, negative affect and paranoia, with no significant differences found, *F*(3, 344) = 0.82, *p* = .482. Consequently, it is unlikely that the predominantly female sample will affect subsequent analyses.

*N* = 91 of the participants (26%) agreed to wear a sleep monitor for 7 nights. As with the main sample, the majority of these participants were female (79%) and ranged in age from 18 to 60 (*M* = 29.88, *SD* = 12.95). MANOVA revealed significant differences between participants who wore, versus did not wear, a sleep monitor, *F*(4, 434) = 4.84, *p* = .001. Examination of the univariate tests revealed that the significant multivariate effect was due to differences in the age of participants who wore versus did not wear a sleep monitor, *F*(1, 437) = 19.24, *p* = .001, and not due to different levels of problems sleeping, affective experiences, or paranoid thinking, *F*(4, 437) ≤ 0.31, *p* ≥ .576. Chi square revealed no significant gender differences between the two groups, *X*^*2*^(1) = 0.43, *p* = .514. In short, the subset of participants who agreed to wear a sleep monitor tended to be younger than those who provided self-report data only (*M* = 29.87 vs. 36.49); however, the samples did not differ in terms of their gender, experiences of insomnia, negative affect, or paranoid thinking. The descriptive statistics for both samples can be seen in Table 1.

**Table 1:** Descriptive statistics for those providing self-report data and those providing objective sleep data.

	Full sample (*N* = 348)			Participants who wore a sleep monitor (*N* = 91)		
Variable	Mean	SD	Min-max	Mean	SD	Min-max^a^
Age	36.49	12.76	17–77	29.87	12.95	17–60
Total insomnia	17.78	5.29	8–32	17.48	5.24	9–30
Sleep onset	8.54	3.04	4–16	8.56	3.13	4–16
Sleep maintenance	9.24	3.08	4–16	8.92	3.06	4–16
Negative affect	12.52	11.42	0–63	12.93	11.97	0–59
Paranoia	18.48	5.8	16–76	18.85	5.23	16–39
Total sleep time^b^ (hours)	-	-	-	6.96	1.01	3.65–9.33
Sleep latency^b^ (minutes)	-	-	-	21.35	14.65	2.6–61.83
Number of awakenings^b^	-	-	-	3.23	2.62	0–11

^a^ Refers to the minimum and maximum score reported by participants

^b^ All objective sleep outcomes are based on mean recordings over 7 nights.

### Procedure

Participants first read an online information sheet detailing the first phase of the research (i.e., the online survey) before indicating their agreement with an electronic consent form. Participants subsequently completed an online questionnaire containing self-report measures of insomnia, negative affect, and paranoid thinking. All participants who completed the online survey were asked if they would like to take part in the second phase of the study that involved using a sleep monitor to objectively monitor their sleep for seven consecutive nights. Participants who expressed an interest arranged a time to meet the researcher who provided information about the study, instructions on how to use the sleep monitor, answered any questions and took written consent from the participants. The Research Ethics Committee in the Department of Psychology at the University of Sheffield granted ethical approval for the research.

### Measures

#### Insomnia

Insomnia (including separate measures of sleep onset and maintenance) was measured using the 8-item insomnia subscale of the Sleep-50 questionnaire [59]. Participants were asked to rate (on a 4-point scale) the extent to which they agree with items such as “I worry so much it prevents me from falling asleep” and “I wake up during the night” with respect to the last 4 weeks. A subsample of the participants were asked to wear a Zeo Sleep Manager that uses a dry fabric, silver coated headband sensor to collect single channel electrophysiological recordings of eye movements, electroencephalography (EEG), and muscle tension. These signals are sent wirelessly from the headband sensor to a small base unit, much like a radio alarm clock, where they are processed and amplified in real time using an artificial neural network. The Zeo Sleep Manager has been validated as an accurate device for measuring sleep patterns in adults when compared against polysomnography and actigraphy [60, 61]. The monitor provided objective measures of total sleep time, sleep latency, and the number of times that each participant woke each night. Mean values for each sleep parameter were calculated over the 7 nights and used in the subsequent analysis.

#### Negative affect

The shortened form of the Depression, Anxiety, and Stress Scale (DASS-21) was used to measure levels of negative affect. Participants were asked to identify (on a 4-point scale) the extent they agreed with items such as “I couldn’t seem to experience any positive feeling at all” and “I felt scared without any good reason”. Participants were asked to consider their responses in light of their experiences over the previous week. The DASS-21 has been found to be a valid and reliable measure of negative affect in both clinical and general population samples and scores on the shortened form have been found to be highly correlated with scores on the longer version of the scale [62, 63].

#### Paranoia

Paranoid thoughts were measured using Part B of the Green Paranoid Thoughts Scale (GPTS-B) [64], that focuses on persecutory thoughts. Participants were asked to indicate the extent to which they agreed or disagreed (on a 5-point scale) that sixteen statements applied to them over the previous month, including “Certain individuals have had it in for me” and “It was difficult to stop thinking about people wanting to make me feel bad”.

### Approach to analysis

The self-report data on levels of insomnia, negative affect and paranoia (*N* = 348) was analysed using SEM. First, an exploratory factor analysis (EFA) was undertaken on one-half of the data, then the resulting factor structure was cross-validated on the other half using confirmatory factor analysis (CFA). This 2-step process allows for a diagnostic stage, where inaccuracies with the outcome measures can be ironed out, followed by a confirmatory phase, where the latent structure is cross-validated on a separate sample before a full path analysis is conducted. This procedure is routinely adopted, reduces measurement error, creates more accurate SEM models, and allows for greater confidence in the resulting analyses [65–68]. Following the exploratory and confirmatory factor analyses, two path models of the relationships between difficulties sleeping, negative affect, and paranoia were evaluated using SEM. The relationship between objective measures of insomnia, negative affect, and paranoia (*n* = 91) was explored separately using linear regression, as the number of participants providing objective sleep data was too low to permit the use of SEM [69].

## Results

### Exploratory factor analysis

EFA was conducted using SPSS v21 [70] in order to understand the latent structure of each scale. In addition, an R-menu (v2.0) developed by Basto and Pereira [71] was used to provide more accurate factor retention methods otherwise not available in SPSS) for a review see, [72]). Direct oblimin rotation was used, which allows the extracted factors to correlate (as we expected them to). The analysis used several methods to decide on the appropriate number of factors to retain including the K1 rule [73], examination of the scree plot [74], parallel analysis [75], Velicer’s minimum average partial (MAP) test [76], the optimal co-ordinates test (a more objective version of the scree plot, [77]), and the comparative data technique [72].

#### The depression, anxiety, stress scale (DASS-21)

One item from the DASS-21, “I was aware of dryness of mouth”, did not correlate with any other items (*r* < 0.30). Therefore, following Field’s [78] recommendations, this item was removed and EFA was applied to the remaining 20-items. Four items were removed as they had substantive cross loadings [i.e. cross loadings greater than 0.36 were deemed substantive according to Steven’s criteria, 79] with other factors leaving 16 items. The Kaiser-Meyer-Olkin measure of sampling adequacy (KMO = 0.92) and Bartlett’s test of sphericity, *X*^2^(120) = 1493.39, *p* < .001, indicated that the data was suitable for principle component analysis (PCA). A clear and simple three factor solution was extracted that explained 63.68% of the variance (see Table 2 for rotated factor loadings). Factor 1 represented levels of depression, Factor 2 represented levels of anxiety, and Factor 3 represented levels of stress. All factors showed good internal reliability (α = 0.92, 0.73, and 0.82, respectively).

**Table 2:** Exploratory factor analysis of the depression, stress, anxiety scale (DASS-21, *N* = 166).

	Rotated factor loadings			
Item	F1: Depression	F2: Anxiety	F3: Stress	Communalities
I felt that I had nothing to look forward to	0.87			0.80
I felt that life was meaningless	0.85			0.66
I was unable to become enthusiastic about anything	0.84			0.75
I couldn’t seem to experience any positive feeling at all	0.79			0.81
I felt I wasn’t worth much as a person	0.78			0.69
I found it difficult to work up the initiative to do things	0.70			0.53
I felt downhearted and blue	0.70			0.64
I was aware of the action of my heart (e.g., sense of heart rate increase)		0.84		0.73
I experienced trembling (e.g., in the hands)		0.63		0.45
I experienced breathing difficulty		0.61		0.53
I felt I was close to panic		0.55		0.63
I found it hard to wind down			0.87	0.72
I found it difficult to relax			0.76	0.71
I was intolerant of anything that kept me from getting on			0.64	0.59
I tended to overreact to situations			0.58	0.54
I was worried about situations in which I might make a fool of myself			0.55	0.44
Cronbach’s alpha	0.92	0.73	0.82	

*Note*: Factor loadings below .36 are not substantive values based on the sample size and have therefore been omitted [79]

#### Sleep-50 insomnia subscale

Correlations between the items in the insomnia subscale of the Sleep-50 questionnaire were within the acceptable range (0.30 < *r* < 0.85), meaning that all of the items were suitable for PCA. Sampling adequacy was verified (KMO = 0.78) and Bartlett’s test of sphericity was significant, *X*^2^(28) = 407.58, *p* < .001. A clear, two factor solution was derived that explained 59.40% of the variance (see Table 3 for rotated factor loadings). The items loading on Factor 1 reflected problems related to sleep onset (the time taken to fall to sleep) while the items loading on Factor 2 reflected problems related to sleep maintenance (time spent asleep after sleep onset). Both factors had good internal reliability (α = 0.77 and 0.74, respectively).

**Table 3:** Exploratory factor analysis of the insomnia subscale of the sleep-50 questionnaire (*N* = 166).

	Rotated Factor Loadings		
Item	Sleep Onset	Sleep Maintenance	Communalities
I worry so much it prevents me from falling asleep	0.91		0.79
I find it difficult to fall asleep	0.86		0.72
I find it hard to relax	0.80		0.63
I sleep too little	0.43		0.35
I wake up during the night		0.80	0.59
I wake up early and cannot get back to sleep		0.74	0.53
After waking up during the night, I fall asleep slowly		0.73	0.69
I sleep lightly		0.67	0.45
Cronbach’s alpha	0.77	0.74	

*Note*: Factor loadings below .36 are not deemed substantive given the sample size and have therefore been omitted [79]

#### Green paranoid thoughts scale–Part B

Correlations between items in Part B of the Green Paranoid Thoughts Scale were within the acceptable range (0.30 < *r* < 0.85), meaning that all of the items were suitable for PCA. Sampling adequacy was verified (KMO = 0.80) and Bartlett’s test of sphericity was significant, *X*^2^(45) = 1027.81, *p* < .001. A single factor solution was obtained comprising of items with factor loadings greater than 0.36 explaining 50.76% of the variance (see Table 4 for rotated factor loadings). These items were parcelled together to create a composite score for paranoia that had good internal reliability (α = 0.88).

**Table 4:** Summary of retained items and communalities after extraction for Part B of the green paranoid thoughts scale (*N* = 166).

Item	Factor Loading	Communalities
People have been hostile towards me on purpose	0.86	0.75
It was difficult to stop thinking about people wanting to make me feel bad	0.82	0.67
I was preoccupied with thoughts of people trying to upset me deliberately	0.81	0.66
I was annoyed because others wanted to deliberately upset me	0.79	0.63
Certain individuals have had it in for me	0.66	0.43
I was angry that someone wanted to hurt me	0.63	0.4
I was distressed by people wanting to harm me in some way	0.63	0.4
I was sure certain people did things in order to annoy me	0.63	0.39
The thought people were persecuting me played on my mind	0.58	0.34
I was distressed by being persecuted	0.55	0.3
Cronbach’s alpha	0.88	

*Note*: Factor loadings greater than .36 were retained and parcelled together to create a composite, single factor representing levels of paranoia [79]

### Confirmatory factor analyses

CFA was conducted using AMOS v22 [80] based on a covariance matrix that consisted of parcelled items derived from the previous exploratory factor analysis. Item parcelling is a commonly used method in SEM, whereby two or more items from each outcome measure are combined (or parcelled) to form an average composite score [81]. For example, the sixteen items from the DASS-21 were combined into four individual parcels representing negative affect (i.e., NA1, NA2, NA3, and NA4). The use of item parcels (rather than individual items) has several advantages, including more stable parameter estimation, reduced impact of idiosyncratic items, fewer parameters to be estimated and thus simplified model interpretation [82–84]. Normality checks revealed that the data was not normally distributed (multivariate kurtosis = 84.92, C.R. = 33.87). This is not uncommon when people report their experiences of problems sleeping, affective experiences, and paranoid thinking as generally, most people have relatively few negative experiences, while a few people report many. Therefore, maximum likelihood estimation was used in combination with bootstrapping procedures drawn from 10,000 samples and item parcelling, a practice that is robust to violations of non-normality in structural equation modelling [84–86].

#### Proposed CFA model

The EFA found a very similar factor structure to previous studies that have sought to validate the DASS-21 [63], in that we extracted factors relating to depression, stress, and anxiety. However, Henry and Crawford specified that these three orthogonal factors together formed a fourth overarching factor—namely, negative affect [62]. We therefore decided to take the same approach by combining the factors representing levels of depression, stress, and anxiety into a single factor representing levels of negative affect. This decision also meant that fewer parameters needed to be estimated in the CFA and subsequent path analysis.

**Fig 1:**
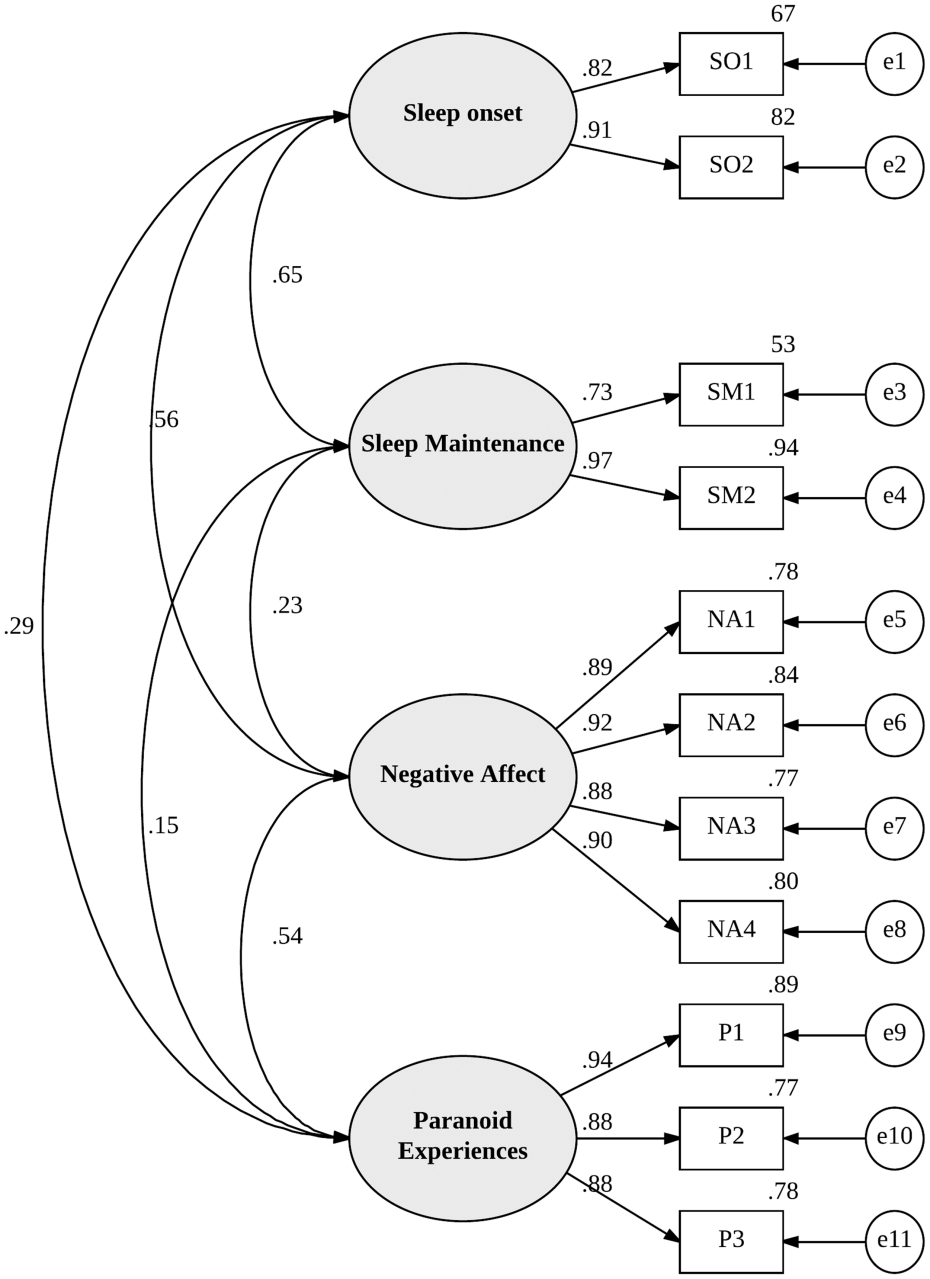
Confirmatory factor analysis. Note: e = error, SO = problems with sleep onset, SM = problems with sleep maintenance, NA = negative affect. P = paranoia. Rectangular boxes represent indicator variables (e.g., items from the DASS-21) that have been parcelled together to form an average (e.g., SO1 and SO2 are parcels consisting of items representing sleep onset), ovals represent latent variables while circles represent error or disturbance.

CFA was conducted on the parcelled items representing problems with sleep onset, sleep maintenance, paranoid thinking, and negative affect. Fig 1 depicts the full model with standardized parameter estimates. The fit indices all indicated an acceptable model fit (CFI = 0.98, RMSEA = 0.08, SRMR = 0.04, CMIN/DF = 2.04), suggesting that the predicted model fitted the observed data. Inspection of the standardized residual covariances further supported the adequate fit of the model. Specifically, these residuals ranged from -0.80 to 1.72 with no values larger than 2.58 indicating no statistically significant discrepancies.

All of the observed variables had high and statistically significant (*p* < .001) loadings on the respective latent variables (Fig 1 shows the magnitude of the factor loadings). In terms of the relationships between the latent variables, there were significant positive relationships between problems with sleep onset and sleep maintenance (*r* = 0.65, 95% CI = 0.53 to 0.75), problems with sleep onset and paranoia (*r* = 0.29, 95% CI = 0.13 to 0.42), as well as between problems with sleep onset and negative affect (*r* = 0.56, 95% CI = 0.43 to 0.67). Problems with sleep maintenance were significantly correlated with negative affect (*r* = 0.23, 95% CI = 0.08 to 0.36), but not with paranoia (*r* = 0.15, 95% CI = -01 to .28). Finally, there was a significant correlation between negative affect and paranoia (*r* = 0.54, 95% CI = 0.37 to 0.69).

#### Path analyses of a partially and fully mediated model

**Fig 2:**
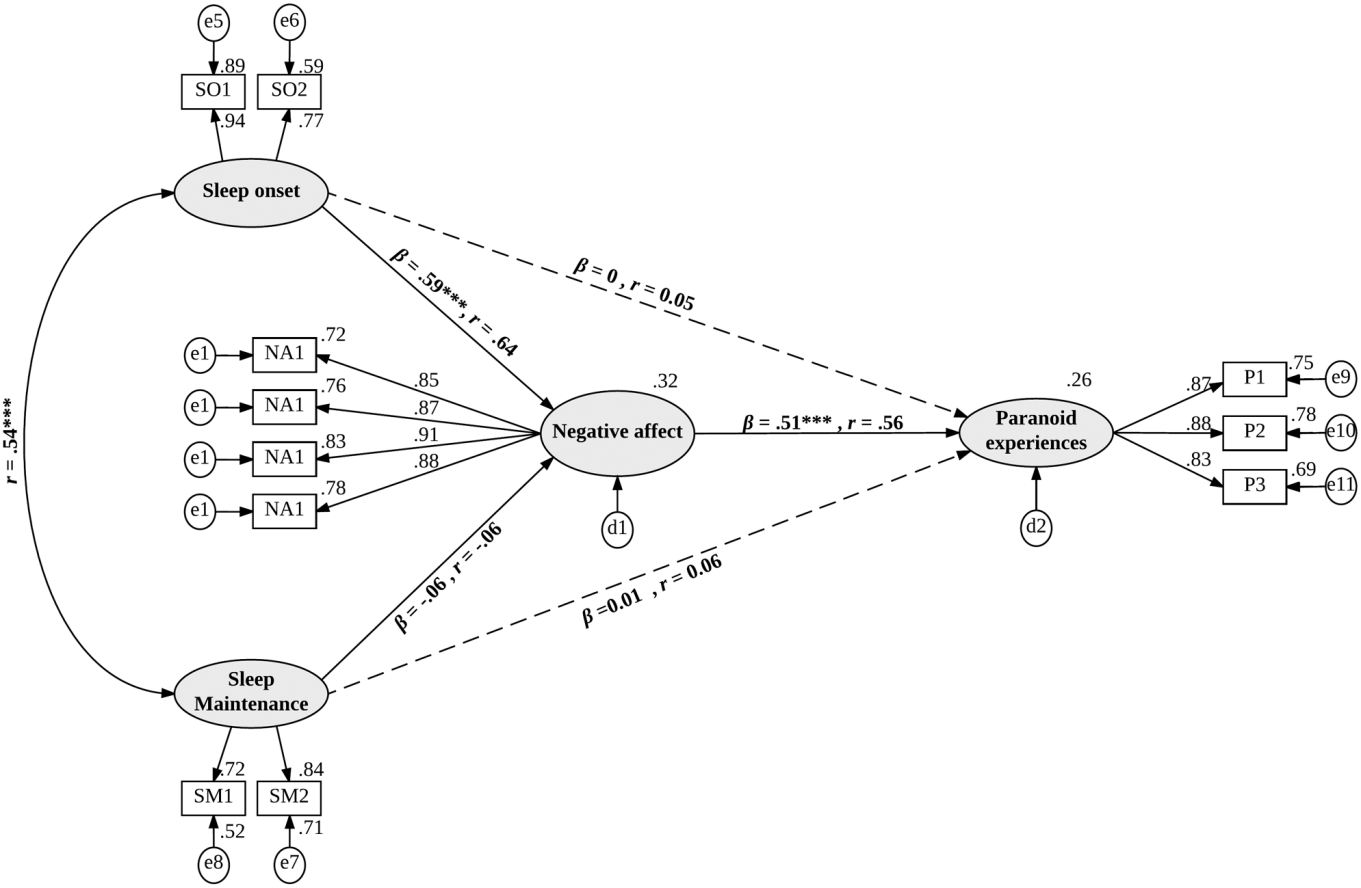
A partially and fully mediated model of the relationship between problems sleeping and paranoid thinking with factor loadings and standardized regression weights. Note: e = error, d = disturbance, SO = sleep onset, SM = sleep maintenance, NA = negative affect. P = paranoia. Rectangular boxes represent indicator variables (e.g., survey responses) which have been parcelled together to form an average (e.g., SO1 and SO2 are parcels consisting of items representing sleep onset), ovals represent latent variables while circles represent error or disturbance. Solid lines represent a fully mediated model; while the addition of the two dashed lines form a partially mediated model.

The final stage of the analysis used SEM based on a covariance matrix to explore the relationships between the variables examined in the preceding CFA. Fig 2 shows the relationship between insomnia and paranoid thinking, either fully or partially mediated by negative affect. The fully mediated model (indicated by the solid lines in Fig 2) specifies that problems with sleep onset and sleep maintenance do not have a direct relationship with paranoia. Instead, the relationship is specified as mediated by negative affect that, in turn, has a direct relationship with paranoia. In the partially mediated model (indicated by the solid lines with the addition of two dashed lines in Fig 2), problems with sleep onset and sleep maintenance have both a direct relationship with paranoia and an indirect relationship via negative affect.

The fully mediated model provided a good fit to the data (CFI = 0.97, RMSEA = 0.07, SRMR = 0.05, CMIN/DF = 2.99) and the standardized residual covariances contained only one value above 2.58. Problems with sleep onset had a significant, indirect relationship with paranoia that was mediated by negative affect (*β* = 0.30, *p* < .001, *r* = 0.35); however, the indirect relationship between problems with sleep maintenance and paranoia via negative affect was not significant (*β* = -0.03, *p* = .385, *r* = 0.-.03). A significant association was found between problems with sleep onset and negative affect (*β* = 0.59, *p* < .001, *r* = 0.64), but not between problems with sleep maintenance and negative affect (*β* = -0.06, *p* = .411, *r* = -0.06), supporting the above relations. As expected, negative affect was significantly and positively related to paranoia (*β* = 0.51, *p* < .001, *r* = 0.56).

The partially mediated model also provided a good fit to the data (CFI = 0.97, RMSEA = 0.08, SRMR = 0.05, CMIN/DF = 3.00) and the standardized residual covariance’s contained no values greater than 2.58. The finding that both fully and partially mediated models provided a good fit to the data is not surprising as these models are derived from the same CFA and are nested within each other. The interesting aspect of this analysis comes from examination of the regression pathways from sleep problems (onset and maintenance) to paranoia. Neither difficulties falling to sleep (sleep onset) nor problems staying asleep (sleep maintenance) were directly associated with paranoia (*β* = 0, *p* = .952, *r* = 0.06 and *β* = 0.01, *p* = .864, *r* = 0.06, respectively). Instead, as can be seen in the fully mediated model (see Fig 2), sleep onset problems, but not sleep maintenance problems, were indirectly associated with paranoia via negative affect. Taken together, these findings support a model in which negative affect fully, rather than partially, mediates the relation between sleep problems and the experience of paranoia.

### Analysis of objective data on sleep difficulties

Table 5 shows the correlations between the self-report (insomnia total, sleep onset, and sleep maintenance) and objective (total sleep time, sleep latency, and the number of awakenings per night) measures of sleep. The correlations tended to be relatively weak (-0.05 < *r* < 0.32), indicating a discrepancy between how participants felt that they slept and how they actually slept. Regression was used to investigate the impact of objective measures of sleep on negative affect and paranoid thinking. Objectively measured sleep latency (the time taken to fall asleep), the average number of awakenings per night (an objective measure of sleep maintenance), and total sleep time did not predict the experience of paranoia (*β*s = -0.14, -0.16, and 0.01, respectively, *p* > .05) or negative affect (*β*s = 0.01, 0.03, and 0.06, respectively, *p* > .05). These findings suggest that perceived, rather than actual, sleep problems are associated with negative affect and the experience of paranoia.

**Table 5:** Correlations between self-report and objective measures of sleep.

	V1	V2	V3	V4	V5	V6
V1. Insomnia total ^a^	1					
V2. Sleep onset ^a^	.85**	1				
V3. Sleep maintenance ^a^	.84**	.44**	1			
V4. Total sleep time^b^	-0.04	-0.01	-0.05	1		
V5. Sleep latency^b^	0.11	.26*	-0.08	.21*	1	
V6. Awakenings per night^b^	.31**	.21*	.32**	-0.03	.34**	1

Note:

* *p* < .05,

** *p* < .01.

^a^ Self-report measure of sleep assessed by the Insomnia Subscale of the Sleep-50 questionnaire [25].

^b^ Objective measure of sleep assessed by the Zeo sleep monitor

## Discussion

The present research addresses a number of issues that are important to understanding the relationship between sleep problems and paranoia, including both practical issues (e.g., relating to measurement) and conceptual issues (e.g., the impact of different types of insomnia and the extent to which levels of negative affect mediate the relationship between difficulties sleeping and paranoid experiences). Specifically, we differentiated between two different facets of insomnia (namely, difficulties with sleep onset and sleep maintenance) and obtained objective measures of sleep from a subset of the sample. Structural equation modelling was then used to simultaneously investigate the relationship between both aspects of insomnia and negative affect and paranoid experiences. The findings suggested that problems initially falling to sleep (sleep onset), but not staying asleep (sleep maintenance), were associated with both negative affect and paranoid thinking. However, problems with sleep onset were not *directly* associated with paranoid thoughts; instead, the effects were mediated by negative affect. Furthermore, these relations only held for perceived sleep (i.e., self-reported measures of sleep) and objective indicators of sleep were not associated with negative affect or the experience of paranoia, suggesting that it is how people believe that they have slept that is the key determinant of outcomes, rather than how they actually slept.

### Insomnia is not directly related to paranoia

The present research found no evidence of a direct relationship between insomnia and the experience of paranoia; instead, the relationship was fully mediated by negative affect. This finding compliments previous research that points to the role of negative affect in the relationship between sleep problems and paranoia. Specifically, research on the interplay between insomnia and emotion (for reviews see, [87, 88]) suggests that insomnia can serve to amplify the effect of negative events. For example, Gujar, Yoo, Hu, and Walker [89] found an enhanced emotional reaction to negative stimuli among participants who were sleep-deprived compared to those who were not. Similarly, Zohar, Tzischinsky, Epstein, and Lavie [90] found that reduced sleep not only amplified the negative emotional consequences of disruptive daytime events but also blunted the positive consequences of rewarding activities. Taken together, these findings suggest that diminished emotional responses to positive events coupled with heightened emotional responses to negative events could provide one explanation as to why insomnia increases negative affect.

In turn, it is also important to understand why negative affect is associated with paranoid thinking, given the strong relationship reported here and elsewhere [34, 37, 91–94]. One possibility is reasoning biases. For example, people with higher levels of stress and anxiety have been shown to be more likely to jump to conclusions than people with lower levels [92, 93] and evidence suggests that anxiety can influence the processing of threat-related stimuli [95]. It is therefore possible that negative affect leads to cognitive and attentional distortions that serve to initiate and/or maintain paranoid thoughts, as described by theoretical models of the formation of paranoid thoughts [20]. Future research might usefully examine these mechanisms using designs that are better able elucidate the proposed mechanisms (e.g., longitudinal designs, experience sampling etc.). For example, recent research comparing a sleep restricted, non-clinical group to a control group (i.e., normal sleep) reported that, not only does sleep restriction lead to significant increases in psychosis like experiences (including paranoia), but that changes in psychosis like experiences are mediated by changes in negative affect and other candidate mechanisms (e.g., cognitive processes) [26].

### The differing impact of difficulties getting to sleep versus difficulties staying asleep

Previous research on the relationship between sleep problems and paranoid experiences has not differentiated between problems falling asleep (sleep onset) versus staying asleep (sleep maintenance). The present research found that only difficulties falling to sleep are associated (indirectly) with paranoid thinking. Problems staying asleep were not associated with either negative affect or the experience of paranoia. This finding has a number of important implications for both past and future research. Specifically, studies that conceptualise and / or measure insomnia as a combination of sleep onset and sleep maintenance difficulties may underestimate the strength of the relationship between insomnia and paranoia by including sleep maintenance in their measurements. Furthermore, interventions designed to reduce negative affect and / or paranoia may wish to consider techniques that specifically address sleep onset difficulties (e.g., sleep restriction, stimulus control etc.) to target the sleep difficulties that are most strongly associated with these outcomes.

To our knowledge, the present research is the first to explore the relationships between different sleep difficulties and paranoid thinking; consequently, there is little empirical data to draw upon in order to understand why problems with sleep onset and maintenance seem to be differentially associated with outcomes. Indeed, the different relationships between problems with sleep onset, maintenance, and negative affect reported here stands in contrast to studies which find that both sleep onset and sleep maintenance problems are related to negative affect [96–100]. For example, Taylor et al. [27] reported that those with combined insomnia (i.e., both onset and maintenance difficulties) were significantly more depressed than those with onset and maintenance insomnia in isolation (although, again, there were no differences between onset and maintenance insomnia). It should be noted, however, that Taylor et al. [27] used clinical cut-off criteria to operationalize sleep onset and sleep maintenance insomnia. In contrast, the present research focused on a general population sample with varying severities of sleep difficulties (indeed, most experienced relatively few difficulties). Indeed, Sheaves et al. [45] reported that problems with sleep onset were more prevalent than problems with sleep maintenance in those classified as at ‘high risk’ of mental health difficulties in a student sample. Consequently, it may be that problems with sleep maintenance are only associated with mental health when those problems are very severe (e.g., among clinical samples). In contrast, problems with sleep onset may be distressing even when less serious.

Alternatively, problems with sleep onset, but not maintenance, may appear to be associated with negative affect and paranoia because some of the items assessing sleep onset difficulties (e.g., “*I worry so much it prevents me from falling asleep*” and “*I find it hard to relax*”) could feasibly assess elements of negative affect, whereas the items measuring sleep maintenance do not contain items that might overlap with affective experiences. Consequently, the differential relationships described in the present research may be due (at least in part) to their measurement. In order to investigate this idea, we removed the two items given as examples from above from the measure of sleep onset and repeated our analyses. Self-reported problems with sleep onset still significantly predicted negative affect (*β* = 0.66, *t*(3, 344) = 5.91, *p* < .001), suggesting that the different relationship between sleep onset problems and sleep maintenance problems and negative affect are unlikely to be explained by the observation that the present measure of difficulties with sleep onset may also have reflected negative emotion. Taken together then, explanations for the relationships between different types of insomnia and negative affect and paranoia warrant further examination before definitive conclusions are drawn.

### Objective measures of sleep were not associated with the experience of paranoia

The present research found no relationship between objective measures of sleep and the experience of negative affect and paranoia. On the one hand this is surprising, given the strength of the relationships found with (aspects of) self-reported sleep in both the current and in previous research. However, there is often a discrepancy between perceived and actual sleep [38–42] and evidence suggests that people have a tendency to overestimate sleep latency and underestimate total sleep time (for a review see [43]. The notion that the perception of sleep, rather than actual sleep, is associated with negative affect and paranoia has important implications, especially for the treatment of insomnia. Specifically, interventions that are able to address specific thought processes that are detrimental to sleep onset are perhaps best placed to improve affective and paranoid experiences. For example, interventions for insomnia based on the principles of CBT (CBTi) might attempt to bring maladaptive thoughts (e.g. “*I must sleep for at least eight hours or I will not be able to function tomorrow*”) more in line with reality (“*even if I sleep for six hours tonight*, *then I will still be able to function well tomorrow*”). This cognitive restructuring can reduce the distress associated with what are often misconceptions about sleep and, in turn, reduce negative affect and, by extension, paranoia.

### The importance of affective experiences

The importance of negative affect in explaining the relationship between sleep problems and paranoia suggests that, in addition to trying to improve sleep (or perceived sleep), interventions might also try to help people to identify and use effective strategies for regulating their emotions. Further support for this idea is provided by evidence that suggests that persecutory ideation and inadequate sleep are both associated with difficulties regulating emotions [11, 101–104]. Fortunately, strategies for regulating emotions (e.g., reappraisal) have been shown to be effective both in shaping emotional experiences [105] and reducing psychopathology [106], suggesting that interventions that combine strategies for dealing with insomnia with strategies for improving emotion regulation may be particularly successful in reducing paranoia.

### Measuring difficulties sleeping, negative affect, and paranoid experiences

The present research conducted analyses to assess the latent structure of the measures. Consequently, it may be helpful to discuss similarities and differences between our findings and extant psychometric assessments of the measures that we employed; namely, of difficulties sleeping (the insomnia subscale of the Sleep-50), negative affect (the DASS-21), and paranoid experiences (the GPTS-B).

To our knowledge, the present study reports the first attempt to assess the latent factor structure of the insomnia subscale of the Sleep-50 [107]. Specifically, to assess difficulties with sleep onset and sleep maintenance in a sample drawn from the general population. Our analyses supported a two-factor solution reflecting difficulties getting to sleep and difficulties staying asleep. Although items such as, “*I sleep too little*”, might reflect both problems with sleep onset and sleep maintenance, it was notable that this item only loaded (significantly) on the factor representing difficulties with sleep onset. However, it did have the strongest cross loading of all items in the insomnia subscale suggesting that future work may want to only include items that definitively reflect problems getting to sleep OR problems staying asleep (but not both).

As with previous validation studies using the DASS-21 [62, 63], the present study found the DASS-21 to be a reliable and valid measure of affective experiences. For example, we found the same clear three factor structure (i.e., reflecting depression, anxiety, and stress) explained by a fourth, overarching category of negative affect as Henry and Crawford [63]. Finally, the present research largely replicated previous research using Part B of the Green Paranoid Thoughts Scale [64] in that it seemed to be a reliable and valid tool for measuring persecutory thinking. However, we could not replicate the three-factor sub-structure of conviction, preoccupation, and distress due to substantial cross loading between these factors. This suggests that, in the present sample at least, the measures intended to reflect the conviction of, preoccupation with, and distress associated with paranoid thoughts are not sufficiently distinct. Consequently, caution may be needed when interpreting sub-domains of this measure, especially when using the GPTS-B in a sample drawn from the general population.

### Limitations and future directions

The present research presents number of novel findings and further elucidates our understanding of the relationships between sleep, negative affect, and paranoia. However, it should be recognised that the research adopted a cross-sectional design and therefore provides limited insight into the causal nature of the relationships between sleep problems, negative affect, and paranoia. While the direction of the putative relations are supported by experimental studies that manipulate, for example, sleep [31, 108–111], the associations described here do not preclude the presence of bidirectional relationships whereby paranoid thinking exacerbates sleep problems and / or negative affect that, in turn, increases paranoid thoughts. Longitudinal research, alongside intervention studies and research employing experience-sampling methods is perhaps best placed to disentangle these potential bidirectional relationships. Some research of this nature is already underway, albeit mainly limited to early pilot work [10, 24, 25, 108, 112].

The findings reported in the present research are also based on a sample of people drawn from the general population; therefore, caution must be taken if seeking to generalise the findings to clinical groups. Specifically, the profile of sleep difficulties in those with psychosis-spectrum diagnoses may be more complicated than simply insomnia alone. For example, psychosis spectrum disorders are associated with a range of sleep problems such as nightmares [113, 114], circadian rhythm problems [115–117], and sleep apnoea [118, 119]. Moreover, difficulties sleeping in clinical groups are often more severe and/or frequent relative to those in healthy controls [1, 3, 114, 115, 120], not to mention that clinical groups are, by definition, likely to have more pronounced paranoid thinking and affective experiences than would be expected in the general population. This wider range of sleep problems and more severe symptoms could feasibly be related in ways that differ to the relationships seen in the present research.

The findings of the present research suggest that problems with (perceived) sleep onset and not sleep maintenance, are associated with paranoid thinking; an effect that is mediated by affective experiences. Future research should aim to elucidate the causal relationships amongst these variables as well as establishing the mechanisms of action (i.e., how and why does sleep influence paranoia?). Studies that can analyse the temporal lag between variables (e.g., experience sampling and longitudinal designs) are perhaps best placed to do so. For example, research that measures aspects of sleep and affective experiences (and other candidate mechanisms) and paranoid thinking at several time points could help to identify covariation and thus how a change in one variable leads to changes in another. Intervention studies, particularly randomised controlled trials (RCTs), could also speak to the causal nature of the relations. For example, a study that is able to successfully improve sleep, and then record subsequent improvement in affective and paranoid experiences, is well placed to inform causality (i.e., an interventionist model, see [121]). A handful of preliminary intervention studies have examined the impact of improving sleep on the experience of paranoia, and have reported mixed results [108, 112]. However, by far the largest RCT on the use of a sleep intervention to improve psychosis like experiences (*N* = 3755) has recently reported that a CBTi intervention delivered to a student population lead to small, but statistically significant effects on paranoid thinking and hallucinations [122]. Furthermore, several studies have shown that improving sleep can improve affective experiences [29, 30, 33, 110, 123]. Taken together, the use of interventions designed to improve sleep in a bid to improve negative affect and paranoia seem warranted.

### Conclusion

The present research provides further evidence on the relationship between sleep difficulties, negative affect, and the experience of paranoia. Specifically, we found that difficulties falling asleep, but not difficulties staying asleep, are associated with negative affect that, in turn, is associated with paranoia. However, these relationships did not hold for objective measures of sleep, suggesting that it is the perception of poor sleep, rather than actual poor sleep, that is associated with negative affect and paranoia. As such, the findings point to a number of potential strategies for reducing paranoia. For example, interventions may profitably target; i) perceived sleep difficulties; ii) sleep onset problems; and / or iii) emotion regulation as a route to reducing negative affect and, thus, paranoid thinking. It is hoped that the present research will stimulate further research in this field, particularly longitudinal and intervention studies, which are needed to disentangle the causal nature of the relationship between insomnia and paranoia.
